# Pulmonary valve replacement for porcelain right ventricular outflow tract following repeated surgical intervention

**DOI:** 10.1186/s44215-024-00126-w

**Published:** 2024-01-12

**Authors:** Satoshi Fujita, Hideki Tatewaki, Ichiro Sakamoto, Yoshihisa Tanoue, Akira Shiose

**Affiliations:** 1 Department of Cardiovascular Surgery, Kyushu University Hospital, 3-1-1, Maidashi, Higashi-Ku, Fukuoka, 812-8582 Japan; 2 Department of Cardiology, Kyushu University Hospital, Fukuoka, Japan

**Keywords:** Adult congenital heart disease, Repeated multivalve surgery, PVR

## Abstract

**Background:**

Repeat surgery is common in adult congenital heart disease, and valve-related procedures are the most frequent indication for re-intervention. In these cases, problems such as advanced adhesion, deterioration and calcification of the prosthesis used, progression of cardiac dysfunction, and worsening of the general condition are often observed.

**Case presentation:**

We herein report a 43-year-old patient with repaired pulmonary atresia and ventricular septal defect who experienced repeated right heart failure and protein-losing enteropathy after multiple bioprosthetic tricuspid and pulmonary valve replacements. The patient was successfully treated with a fourth pulmonary valve replacement and third tricuspid valve replacement using a mechanical valve. During surgery, peeling off and removing the right ventricular outflow conduit was risky due to dense adhesion to the ascending aorta with extremely severe calcification; thus, the mechanical pulmonary valve was implanted to a more proximal position of the right ventricular outflow tract after removing the leaflet only and leaving the stent of the bioprosthetic valve within the conduit. The right heart failure and protein-losing enteropathy were relieved with this surgery, and the patient has remained in remission for over 5 years.

**Conclusion:**

Although severe adhesion and porcelain-like calcification caused by multiple surgical interventions were a major issue in this case, good surgical results were obtained. This method has a major advantage over conventional pulmonary valve replacement with right ventricle outflow tract reconstruction when the right ventricular outflow tract conduit shows severe adhesion and calcification.

## Background

The survival and quality of life of children with congenital heart disease (CHD) have greatly improved with advances in diagnosis and treatment. Most patients with CHD now survive to adulthood [1]. To establish a normal or nearly normal physiology, surgical intervention is performed for most cases of CHD. However, many patients with CHD require multiple surgical interventions throughout their lifetime [1, 2]. Valve-related procedures are the most frequent indication for re-intervention in adult congenital heart disease (ACHD) [2], with multivalve surgery required in a quarter of cases [1]. In cases requiring repeated surgical intervention, problems such as advanced adhesion, deterioration and calcification of the prosthesis used, progression of cardiac dysfunction, and worsening of the general condition are often observed. The number of re-interventions in ACHD will continue to rise as the ACHD population increases.

## Case presentation

A 43-year-old woman (height 145 cm, weight 36.4 kg, body surface area 1.22 m^2^) had been diagnosed with pulmonary atresia and ventricular septal defect. She had received a left original Blalock-Taussig shunt at 1 year old and undergone a Rastelli-type procedure using a bioprosthetic valved conduit at 6 years old. Following a corrective procedure, she underwent right ventricular outflow (RVOT) conduit replacement with a bioprosthetic valved conduit twice, and tricuspid valve replacement (TVR) twice with a bioprosthetic valve for the progression of conduit stenosis and structural valve deterioration (SVD) in her clinical course. The most recent RVOT conduit replacement had been performed with a 25-mm Carpentier Edwards porcine valved conduit (Edwards Lifesciences, Irvine, CA, USA) at 23 years old. Most recently, she had undergone TVR with a 27-mm Carpentier Edwards porcine valve (Edwards Lifesciences) at 33 years old. In total, she underwent open-heart surgery four times with median sternotomy. In addition, she had undergone pacemaker implantation trans-venously with an AAI system for sick sinus syndrome at 33 years old.

Regarding her cardiac function and general condition, right heart failure and protein-losing enteropathy (PLE) developed around 30 years old, resulting from SVD of the tricuspid position. Although PLE was temporarily relieved by re-TVR at 33 years old, right heart failure and recurrent PLE developed due to SVD of the tricuspid position again from around 39 years old. Despite intensive medical therapy, her symptoms and PLE showed repeated exacerbation and remission. At that point, the mean pressure gradient (PG) between the right atrium (RA) and right ventricle (RV) was 10 mmHg, mean RA pressure was 16 mmHg, and tricuspid regurgitation grade was moderate. Regarding the bioprosthetic pulmonary valve, deterioration was also progressing (peak PG, 25 mmHg; regurgitation grade: moderate to severe). The cardiac index (CI) was 2.4 L/min/m^2^, and the systemic cardiac function was generally preserved.

Therefore, we decided to perform a third TVR and fourth pulmonary valve replacement (PVR) using a mechanical valve at 43 years old. In addition, we were concerned about the high risk of developing advanced atrioventricular block in this patient and decided to upgrade the pacemaker to a DDD system with the use of epicardial pacemaker leads. Although intensive and maximum medical therapy was required for a relatively long period due to postoperative right heart failure, relief from PLE and a favorable postoperative course were achieved. The patient has been doing well with maintained serum albumin levels and no recurrence of PLE for over 5 years.

### Surgical procedures

**Fig. 1:**
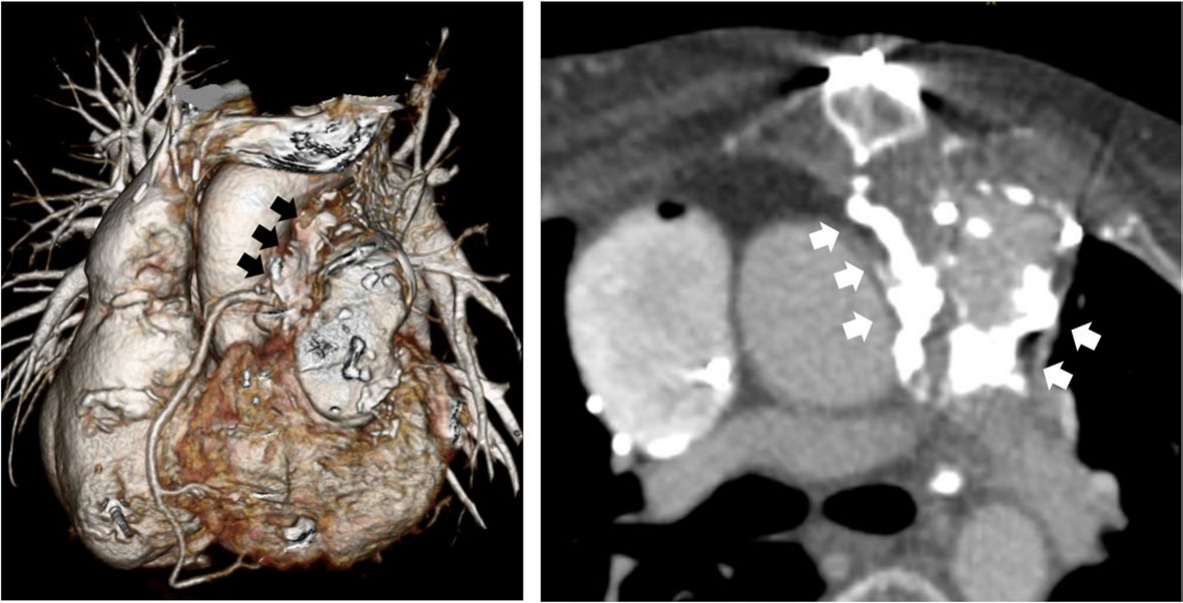
Preoperative computed tomography. The black and white arrows indicate severe calcification around the conduit

**Fig. 2:**
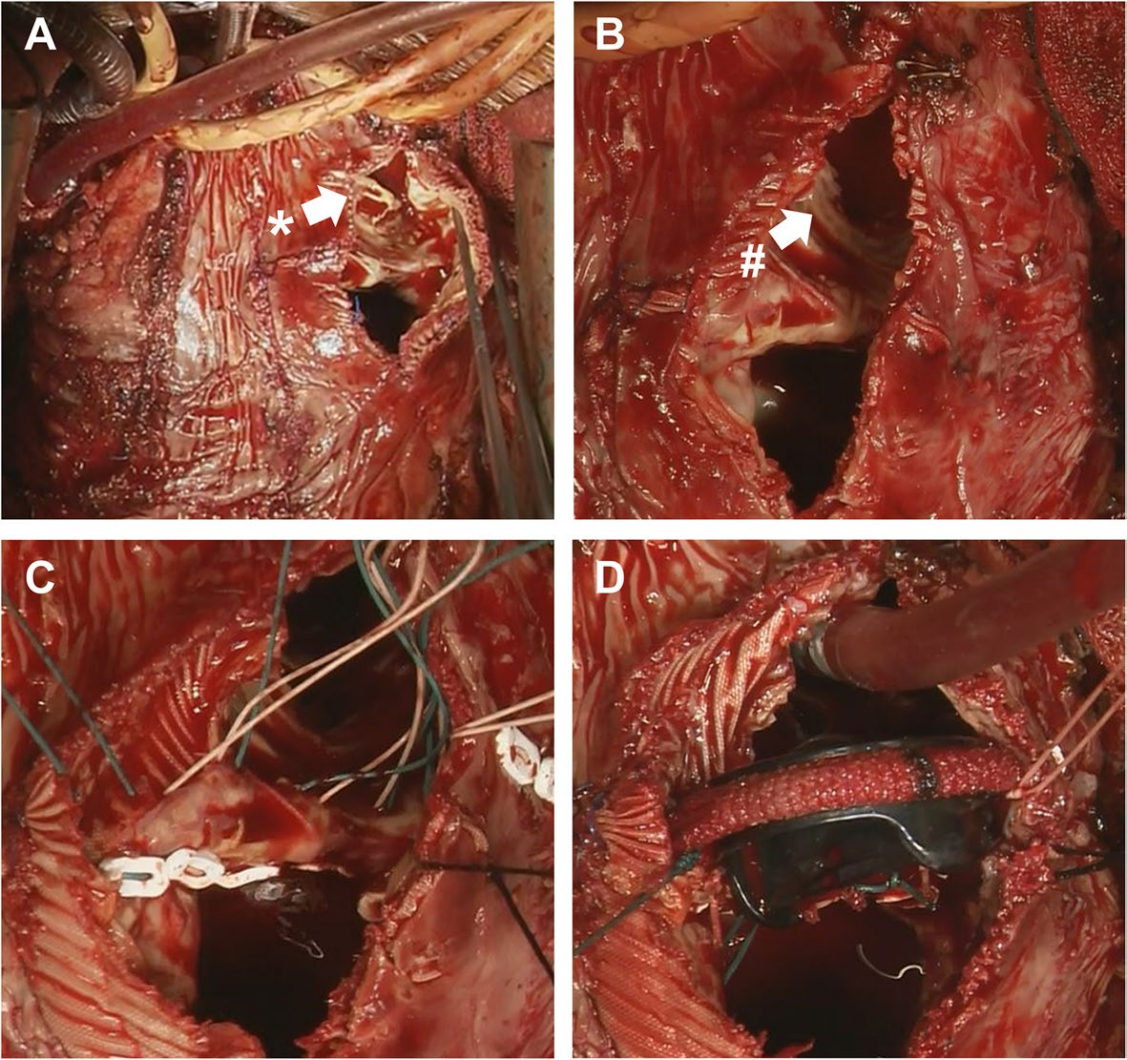
Intraoperative images of the RVOT. **A**, **B** The leaflets were highly calcified and thus were excised and removed, leaving the stent. **C**, **D** The prosthetic valve was implanted in a more proximal position of the RVOT. *The bioprosthetic valve. #The stent left in the conduit. *RVOT* right ventricular outflow tract

Following the fifth redo-sternotomy and adhesiolysis, TVR and PVR were performed using conventional cardiopulmonary bypass (CPB) with central cannulation. All procedures were performed with the heart beating. Following CPB establishment, the deteriorated bioprosthetic valve of the tricuspid position was removed, and TVR using a 27-mm bi-leaflet mechanical valve (St. Jude Medical, St. Paul, MN, USA) was performed via right atriotomy. Subsequently, PVR was performed via a longitudinal incision of the RV free wall, and extended in the RVOT conduit, which showed porcelain-like calcification. As predicted, peeling off and removing the RVOT conduit was risky due to severe calcification and adhesion around the conduit (Fig. 1); thus, we decided to perform PVR after only removing the bioprosthetic valve leaflet, leaving the stent within the conduit. The highly calcified leaflets (Fig. 2A) were excised and removed as much as possible (Fig. 2B). The minimum inner diameter of the remaining conduit with the remnant stent was 21 mm, which was considered sufficient to prevent RVOT stenosis in this patient. In addition, the chest wall was near, creating a space issue inside the RVOT. Thus, a 21-mm bi-leaflet mechanical valve (St. Jude Medical) was selected and implanted in the RVOT, more proximal than the anastomotic site of the RV and conduit (Fig. 2C). To avoid injury of the first septal branch of the left anterior descending coronary artery, valve sutures on the posterior wall were placed on the border line between Dacron graft and native RV wall. In addition, everting matless suture was selected to prevent dehiscence of the valve due to an unsecure valve suture. We paid attention to the valve angle to ensure it was orthogonal to the blood flow axis (Fig. 2D). The incised conduit, RV free wall, and mechanical valve were covered with a patch trimmed from a woven Dacron graft. On the anterior side of the mechanical valve, a mattress suture was placed from the inside to the outside through the patch to secure the valve. Finally, the pacemaker was upgraded to a DDD system by implanting an epicardial lead near the apex before chest closure.

## Discussion and conclusions

Most initial biventricular repair for CHD is corrective in terms of normalizing the cardiac physiology. However, these “corrective” operations are often viewed as palliative because many require surgical re-intervention in the future [1, 2]. Multivalve surgery is not uncommon in ACHD, and surgery on two or more valves at re-intervention is reportedly required in a quarter of these patients [1]. Determining the appropriate timing of re-intervention and having the correct knowledge of anatomical differences in CHD are necessary to achieve successful valve repair and replacement in ACHD.

In our case, the fourth sternotomy procedure before this surgery resulted in extremely severe adhesion in the mediastinum. A previous study reported the performance of PVR without median sternotomy, avoiding re-sternotomy with less extensive adhesiolysis [3]. This method has a major advantage in simple PVR cases but was not applicable in our case due to severe adhesion and calcification around the RVOT conduit and the need for TVR. Peeling off and removing the RVOT conduit was considered risky, so we performed PVR after removing the leaflet only and left the stent of the bioprosthetic valve within the conduit. This procedure requires that no stenosis remains in the remnant RVOT conduit. Because a bioprosthetic valved conduit of a sufficient size had been used at the previous surgery, we were able to perform this procedure without postoperative RVOT stenosis. In addition, the prosthetic valve will inevitably be implanted in the more proximal position of the RVOT. Thus, it is necessary to check whether or not a sufficient space can be secured for prosthetic valve implantation at an angle orthogonal to the blood flow axis and the positional relationship with the coronary arteries [4]. We consider this method able to be applied to homografts with progression of strong calcification.

Regarding the prosthetic valve selection, we chose a mechanical valve in this case. A bioprosthetic valve has advantages in that anticoagulation is unnecessary, and a future trans-catheter valve-in-valve procedure may be possible. However, we decided to use a mechanical valve to avoid the need for further surgical valve re-intervention, repeated operative stress, and SVD over time, which can affect the cardiac function and general condition. Although mechanical valves have issues with regard to anticoagulation, we sometimes choose mechanical valves in patients who have undergone multiple prior surgeries and need to avoid further surgical intervention or who require valve surgery on the left side. We considered a mechanical valve the better option for ensuring a good quality of life in this patient. Excellent surgical results of mechanical PVR with low re-intervention rates under proper anticoagulation and monitoring have been reported [4, 5]. A younger age, longer interval between the repair of congenital defect and PVR, and concomitant surgery are reported as predictors of reoperation [5]. Regarding prosthetic valves at the tricuspid position, the risk of thromboembolism and bleeding is reportedly higher with mechanical valves than with bioprosthetic valves; however, mechanical valves are superior to bioprosthetic valves for avoiding reoperation. The outcomes of mechanical TVR were comparable with those of bioprosthetic TVR in terms of the long-term survival and valve-related events over 15-year postoperative follow-up [6]. Points to note, one of the major limitations of using mechanical prosthesis on tricuspid position is that the patient is no longer able to receive a trans-venous pacemaker implant in the future. A sufficient consideration before mechanical TVR is important, and if deemed necessary, implantation of epicardial pacemaker leads should be performed concomitantly.

Regarding the general condition, PLE was relieved and exacerbated repeatedly in our patient due to SVD progressing over time. PLE is a life-threatening complication that occurs in Fontan patients, with a reported prevalence of 3–15% [7]. Hemodynamic characteristics of Fontan circulation, which include high central venous pressure and low cardiac output, may be the major causes of PLE [7]. However, PLE also occurs in some cardiac patients without Fontan circulation, including those with constrictive pericarditis or chronic congestive heart failure. In PLE patients without Fontan circulation, it has been pointed out that diastolic dysfunction of the RV can be one reason for developing PLE and also be a treatment target, and these patients may have a greater chance of remission from PLE than others because of the high prevalence of possibly treatable responsible lesions, unlike Fontan patients [8]. In our patient, the third TVR and fourth PVR using a mechanical valve provided relief from PLE.

In conclusion, multivalve surgery using a mechanical valve helped relieve PLE and resulted in a favorable postoperative course for our patient undergoing repeated cardiac surgery. Although severe adhesion and porcelain-like calcification caused by multiple surgical interventions were major issues, good surgical results were obtained using a surgical technique that involved removing the leaflet only and leaving the stent of the bioprosthetic valve in the RVOT conduit.

## Data Availability

Data sharing is not applicable to this article, as no datasets were generated or analyzed during the current study.

## Abbreviations

ACHD: Adult congenital heart disease
CHD: Congenital heart disease
CI: Cardiac index
CPB: Cardiopulmonary bypass
PG: Pressure gradient
PLE: Protein-losing enteropathy
PVR: Pulmonary valve replacement
RA: Right atrium
RV: Right ventricle
RVOT: Right ventricular outflow tract
SVD: Structural valve deterioration
TVR: Tricuspid valve replacement
